# IncI1 Plasmid Associated with *bla*_CTX-M-2_ Transmission in ESBL-Producing *Escherichia coli* Isolated from Healthy Thoroughbred Racehorse, Japan

**DOI:** 10.3390/antibiotics9020070

**Published:** 2020-02-07

**Authors:** Eddy Sukmawinata, Ryoko Uemura, Wataru Sato, Shuya Mitoma, Takuya Kanda, Masuo Sueyoshi

**Affiliations:** 1 Graduate School of Medicine and Veterinary Medicine, University of Miyazaki, Miyazaki 889-1692, Japan; eddyswinata@gmail.com (E.S.); matomi1510@gmail.com (S.M.); kanda0823@gmail.com (T.K.); a0d802u@cc.miyazaki-u.ac.jp (M.S.); 2 Department of Veterinary Sciences, Faculty of Agriculture, University of Miyazaki, Miyazaki 889-2192, Japan; wataru9356@gmail.com; 3 Center for Animal Disease Control, University of Miyazaki, Miyazaki 889-2192, Japan

**Keywords:** *E. coli*, horse, extended spectrum β-lactamase, IncI1 plasmid

## Abstract

In our previous study, extended spectrum β-lactamase (ESBL)-producing *Escherichia coli* (ESBLEC) were isolated from healthy Thoroughbred racehorse feces samples in Japan. Some ESBL genes were predicted to be located on the conjugative plasmid. PCR-based replicon typing (PBRT) is a useful method to monitor and detect the association of replicons with specific plasmid-borne resistant genes. This study aimed to evaluate the plasmid replicon associated with ESBLEC isolated from healthy Thoroughbred racehorses at Japan Racing Association Training Centers in Japan. A total of 24 ESBLECs isolated from 23 (10.8%) individual Thoroughbred racehorse feces samples were used in this study. ESBL gene transfer was performed using a conjugation assay. Then, replicon types of ESBLEC isolates and their transconjugants were determined using PBRT. Pulsed-field gel electrophoresis (PFGE) was performed to look at the clonality of the ESBLECs isolates. ESBLECs were detected from 10.8% of healthy Thoroughbred racehorses. The *bla*_CTX-M-2_ was identified as the dominant type of ESBL gene, followed by *bla*_CTX-M-1_ and *bla*_TEM-116_. In this study, only the *bla*_CTX-M-2_ and the IncI1 plasmid were transferred to transconjugants. The PFGE results showed that ESBL genes were distributed in diversity of ESBLECs. This finding suggested that the IncI1 plasmid was associated with the dissemination of *bla*_CTX-M-2_ in Thoroughbred racehorses in Japan.

## 1. Introduction

The emergence of resistance to third-generation cephalosporin, mediated mainly by extended spectrum β-lactamase (ESBL), has become a major health problem [1]. CTX-M is the largest group of ESBL, which has disseminated globally and increased in prevalence since 2000 [2], after the spread of TEM and SHV that were predominant in the 1990s [1,3]. CTX-M types have been grouped into five clusters (CTX-M-1, CTX-M-2, CTX-M-8, CTX-M-9, and CTX-M-25) and over 172 CTX-M types have been reported [4]. This massive worldwide dissemination has been described as the “CTX-M pandemic” [5]. The rapid spread of ESBL genes is mostly due to their location on the plasmid and the ease of transmission between bacteria [5,6,7,8]. Another reason for this increase is the co-resistance phenomenon to other antibiotics, especially to aminoglycosides and fluoroquinolones in CTX-M-producing bacteria, which might facilitate co-selection processes [5].

As a potentially zoonotic bacteria, ESBL-producing *Escherichia coli* (ESBLEC) have received special attention in the area of equine medicine [9]. Most studies of ESBLEC in horses refer to clinical isolates [7], where healthy horses are also potential reservoirs of ESBL-producing bacteria. PCR-based replicon typing (PBRT) is a useful method to monitor and detect the association between replicons and specific plasmid-borne resistance genes, as well as detecting the mobilization capability of resistance genes among different plasmids [10,11]. Incompatibility (Inc) groups F, A/C, L/M, I1, HI2, and N were reported as the predominant plasmid replicon type in antibiotic-resistant *Enterobacteriaceae* isolated from humans and animals [12].

In our previous study, ESBLECs were isolated from 8.2% of healthy Thoroughbred racehorses in Japan Racing Association (JRA) Training Centers in Japan. CTX-M-2 was identified as the most prevalent type of ESBL and some of these were predicted to be located on a conjugative plasmid [9]. The aim of this study was to evaluate the diversity of plasmid replicon types of ESBLEC isolated from healthy Thoroughbred racehorses in Japan. Furthermore, plasmid replicon types were compared between donor and transconjugant isolates in order to evaluate the plasmid replicon types associated with the ESBL gene. 

## 2. Results

### 2.1. Characterization of ESBLEC

In this study, 24 ESBLECs isolated from 23 (10.8%) individual Thoroughbred racehorse feces samples were identified genetically between April 2017 and August 2018. The ESBLEC harboring *bla*_CTX-M-2_ was detected in 87.5% (21/24) of isolates, followed by *bla*_CTX-M-1_ (8.3%; 2/24) and *bla*_TEM-116_ (4.2%; 1/24). All isolates (100%; 24/24) were phenotypically confirmed for ESBL production. None of the isolates were positive for AmpC β-lactamase production. Co-resistance to STX (66.7%; 16/24), SM (50%; 12/24), TC (20.8%; 5/24) and OTC (20.8%; 5/24) was observed. Multidrug-resistant (MDR) ESBLECs were identified from 45.8% (11/24) of isolates showing resistance to at least three classes of antibiotic. 

**Figure 1:**
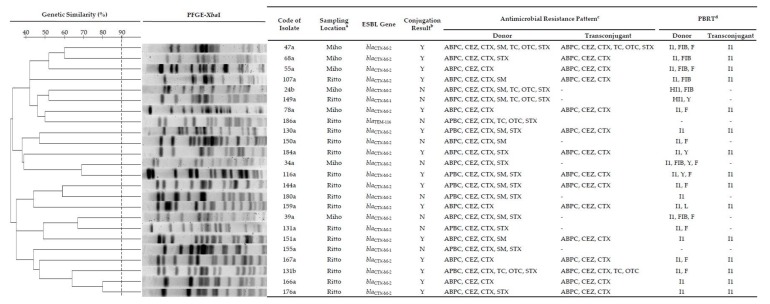
Characterization of extended spectrum β-lactamase (ESBL)-producing *E. coli* (ESBLECs) isolated from healthy Thoroughbred racehorses and their ability to transfer the ESBL gene. ^a^ Miho, Miho Training Center, Japan Racing Association, Ibaraki; Ritto, Ritto Training Center, Japan Racing Association, Shiga. ^b^ Y, Successful conjugation; N, Not successful conjugation. ^c^ ABPC, ampicillin; CEZ, cefazolin; CTX, cefotaxime; SM, streptomycin; TC, tetracycline; OTC, oxytetracycline; STX, trimethoprim-sulfamethoxazole. Antimicrobial susceptibility test was interpreted using CLSI criteria and JVARM report. ^d^ PBRT, PCR-based replicon typing.

PBRT showed typing for 91.7% (22/24) of isolates. The plasmid replicons could not be characterized for 8.3% (2/24) of isolates. The IncI1 plasmid was highly distributed among ESBLEC isolates where the FIB, F, HI1, Y, and L groups were also detected. All ESBLECs investigated were genotypically diverse, as shown by a variety of PFGE patterns. Genetic similarity, ESBL type, antimicrobial resistance pattern and PBRT are summarized in Figure 1.

### 2.2. ESBL Gene Transfer

Conjugation assays were successful in 62.5% (15/24) of ESBLEC harboring *bla*_CTX-M-2_ isolates. The *bla*_CTX-M-1_ and *bla*_TEM-116_ were not conjugated under our experimental conditions. Plasmid transmission to the recipient strains was only shown by the IncI1 plasmid. Two transconjugants were identified phenotypically to have co-resistance with tetracycline derivates and/or STX. The horizontal transmission is demonstrated in Figure 1.

## 3. Discussion

Horses can serve as a natural reservoir of antibiotic-resistant microorganisms, a characteristic which has implications on the health, treatment efficiency and epidemiological safety of people working in close contact with horses [13]. Racehorses have been described as potential reservoirs of ESBLECs in Japan [9]. In the current study, we evaluated the horizontal transmission of ESBLECs detected from 10.8% of samples collected from healthy Thoroughbred racehorses at JRA Training Centers. The *bla*_CTX-M-2_ was identified as the dominant type of ESBL gene, followed by *bla*_CTX-M-1_ and *bla*_TEM-116_. Multidrug-resistance was detected in 45.8% (11/24) of ESBLEC isolates.

In our experimental conditions, *bla*_CTX-M-2_ could be transferred to the recipient transconjugants, but neither *bla*_CTX-M-1_ nor *bla*_TEM-116_ were conjugated. Nevertheless, all of these genes have been reported to be present on conjugative plasmids in previous studies [14,15,16,17]. In addition, *bla*_TEM-116_ has not been reported in humans and animals in Japan, but was recently detected from *Pseudomonas* spp. isolated from fresh vegetables [18]. On the other hand, these ESBL genes were distributed in a range of *E. coli* isolates based on PFGE results.

Plasmid harboring AMR genes have largely been described on typical Inc group plasmids and few have been reported from unidentified group plasmids [19]. The investigation of plasmids is helpful in understanding the dissemination of ESBL genes [20]. Here, we investigated the transferable plasmid associated with *bla*_CTX-M-2_ genes by PBRT. The IncI1 plasmid was distributed to nearly all ESBLEC isolates and only the IncI1 plasmid was capable of transferring to transconjugants in this work. The dissemination of ESBL genes is reportedly mediated by the IncI1 plasmid [21]. *E. coli* was also reported to maintain the IncI1 plasmid without antibiotic selection pressure [22]. Thus, our findings suggested that the IncI1 plasmid has an important role in the spread of *bla*_CTX-M-2_ in racehorses. In contrast to previous studies in Japan, *bla*_CTX-M-2_ located on the IncT plasmid was reported from *K. pneumoniae* isolated from bovine mastitis and *Proteus mirabilis* isolated from humans [23,24]. In a European study, the IncI1 plasmid harboring *bla*_CTX-M-1_ was commonly identified in animal isolates [25]. The IncI1/ST3 plasmids carrying the *bla*_CTX-M-1_ gene are reportedly disseminated in several animal species, including horses in France [26]. In Belgium, *bla*_CTX-M-2_ was found in IncHI1 and IncFIB plasmids from the *E. coli* of horses and showed co-transferred non β-lactam antibiotics [27].

In this study, co-transferred non β-lactam resistance was shown in two *bla*_CTX-M-2_-positive transconjugants harboring the IncI1 plasmid to tetracycline and/or STX (Figure 1). The IncI1 plasmid is often associated with MDR [28]. In Denmark, the IncI1 plasmid carrying *bla*_CTX-M-1_ reported from *E. coli* isolated from dogs was co-transferred with STX [29]. The plasmid harboring the ESBL gene containing resistance to other antibiotics may also be co-selected by non-β-lactam antibiotics [30]. 

In conclusion, the IncI1 plasmid was associated with the dissemination of *bla*_CTX-M-2_ in Thoroughbred racehorses. This information is very useful for the control of the dissemination of *bla*_CTX-M-2_ among the racehorse population. Further analysis such as plasmid subtyping or whole genome sequencing is needed to assess how the *bla*_CTX-M-2_ genes are spreading throughout racehorses in Japan. 

## 4. Materials and Methods 

### 4.1. ESBLEC Isolates

Twenty-four isolates of ESBLEC were studied. These isolates were derived from 23 healthy Thoroughbred racehorse feces samples in JRA Training Centers in Japan. Thirteen isolates were selected from ESBLEC collection isolates, which were isolated from 12 of 147 Thoroughbred racehorse feces samples in a previous study [9]. Additional non-repetitive isolates (one isolate, one horse) were collected from 65 feces samples from the JRA Ritto Training Center between May and August 2018. A total of 212 racehorse feces samples were investigated between April 2017 and August 2018 in this study. The isolation of ESBLEC was as described in a previous study [9].

All isolates were confirmed for the ESBL phenotype using an AmpC and ESβL Detection Set (D68C) Kit, and the results were interpreted based on the manufacturer’s guidelines (Mast Diagnostics, Merseyside, UK). All ESBLEC isolates were stored frozen in TSB with 20% glycerol at −80 °C for further analysis.

### 4.2. Antimicrobial Susceptibility Test

All isolates were tested for susceptibility to twelve antimicrobial agents belonging to seven classes of antibiotic, β-lactam (ampicillin [ABPC], cefazolin [CEZ], cefotaxime [CTX]), aminoglycoside (gentamicin [GM], streptomycin [SM]), tetracycline (tetracycline [TC], oxytetracycline [OTC]), amphenicol (chloramphenicol [CP]), polypeptide (colistin [CL]), quinolone (nalidixic acid [NA], enrofloxacin [ERFX]) and folate antagonist-sulfonamide (trimethoprim- sulfamethoxazole [STX]), by determining the minimum inhibitory concentration (MIC) of these antibiotics based on recommendations from the Clinical Laboratory Standard Institute guidelines [31]. In the case of SM, for which there are no CLSI breakpoints, the results were interpreted based on a report from the Japanese Veterinary Antimicrobial Resistance Monitoring (JVARM) system [32]. Isolates that showed resistance to at least three classes of antimicrobial were considered to have multidrug resistance (MDR) [33].

### 4.3. ESBL Gene Transfer

The transfer of ESBL genes was studied using a conjugation assay for all ESBLEC isolates. A plasmid free and nalidixid acid-resistant (F^-^, Na^r^) of *E. coli* DH5α (Takara Bio Inc., Shiga, Japan) was used as the recipient strain, while all the ESBLECs sensitive to NA served as donors. Conjugation experiments were performed based on our previous study [9]. All transconjugants were confirmed by PCR for genes encoding ESBL production and tested for susceptibility to the same antibiotic used against the donor isolates.

### 4.4. PCR-Based Replicon Typing (PBRT)

The replicon types of ESBL-producing bacteria (donor) and their transconjugants were determined using a previous report [10]. Briefly, amplification by PCR was performed with 18 pairs of primers recognizing HI1, HI2, I1-Ic, X, L/M, N, FIA, FIB, W, Y, P, FIC, A/C, T, FIIAs, F, K, and B/O in five multiplex and three simplex reactions.

### 4.5. Pulse Field Electrophoresis Gel

Pulsed-field gel electrophoresis (PFGE) was carried out as described previously [15]. DNA fragments were separated for 20 h at 14 °C on 1% pulse-field certified agarose gel (Bio-Rad Laboratories, Hercules, CA, USA) in the 0.5× Tris/Borate/EDTA buffer, with a switch ramp time from 6.8 to 35.4 s at a 120° angle, using a CHEF-DRII system (Bio-Rad Laboratories, Hercules, CA, USA). A dendrogram analysis of band-based PFGE patterns was performed using a gene profiler (Scanalytics, Buckinghamshire, UK). A similarity matrix was calculated using the Dice coefficient and cluster analysis was performed using the Unweighted Pair Group Method with Arithmetic mean UPGMA algorithm. A cluster was defined based on a similarity cut-off of 90% with 1.0% optimization and 2.0% band tolerance.
